# The evolutionary impact of androgen levels on prostate cancer in a multi-scale mathematical model

**DOI:** 10.1186/1745-6150-5-24

**Published:** 2010-04-20

**Authors:** Steffen E Eikenberry, John D Nagy, Yang Kuang

**Affiliations:** 1 Department of Mathematics and Statistics, Arizona State University, Tempe, AZ 85287, USA; 2 Current address: Keck School of Medicine, University of Southern California, Los Angeles, CA 90033, USA; 3 Department of Biology, Scottsdale Community College, Scottsdale, AZ 85256, USA

## Abstract

**Background:**

Androgens bind to the androgen receptor (AR) in prostate cells and are essential survival factors for healthy prostate epithelium. Most untreated prostate cancers retain some dependence upon the AR and respond, at least transiently, to androgen ablation therapy. However, the relationship between endogenous androgen levels and cancer etiology is unclear. High levels of androgens have traditionally been viewed as driving abnormal proliferation leading to cancer, but it has also been suggested that low levels of androgen could induce selective pressure for abnormal cells. We formulate a mathematical model of androgen regulated prostate growth to study the effects of abnormal androgen levels on selection for pre-malignant phenotypes in early prostate cancer development.

**Results:**

We find that cell turnover rate increases with decreasing androgen levels, which may increase the rate of mutation and malignant evolution. We model the evolution of a heterogeneous prostate cell population using a continuous state-transition model. Using this model we study selection for AR expression under different androgen levels and find that low androgen environments, caused either by low serum testosterone or by reduced 5*α*-reductase activity, select more strongly for elevated AR expression than do normal environments. High androgen actually slightly reduces selective pressure for AR upregulation. Moreover, our results suggest that an aberrant androgen environment may delay progression to a malignant phenotype, but result in a more dangerous cancer should one arise.

**Conclusions:**

The model represents a useful initial framework for understanding the role of androgens in prostate cancer etiology, and it suggests that low androgen levels can increase selection for phenotypes resistant to hormonal therapy that may also be more aggressive. Moreover, clinical treatment with 5*α*-reductase inhibitors such as finasteride may increase the incidence of therapy resistant cancers.

**Reviewers:**

This article was reviewed by Ariosto S. Silva (nominated by Marek Kimmel) and Marek Kimmel.

## Background

By the time Theodosius Dobzhansky famously claimed that "nothing in biology makes sense except in the light of evolution" [1], cancer was already becoming an excellent illustration of his point. In fact, two decades before Dobzhansky's claim appeared in print, Law [2] demonstrated that, at least in a mouse model, leukemia develops resistance to folic acid antagonists by natural selection, not accommodation. By the mid 1970s the significance of evolution in tumor progression, pathogenesis, and treatment resistance was maturing [3], with interest in evolutionary oncology exploding the in the 1990s [4-10]. Now, cancer is increasingly understood to be an evolutionary phenomenon, although the conditions for evolution by natural selection have been demonstrated unequivocally in only one neoplasm--Barrett's esophagus [11].

Until recently it was an open question whether insights generated by an evolutionary perspective could be translated to the oncology clinic. However, in the last 10 years a wide variety of potential applications has arisen. Evolutionary models of neoplasia led Gatenby, Gawlinsky and colleagues [12-14] to hypothesize that tumor-associated tissue acidosis may be caused by natural selection favoring glycolytic cells in developing carcinomas. Selection for this property, in turn, facilitates invasion of surrounding tissue. Agent-based models studied by Maley and colleagues [15,16] suggest a novel treatment approach--altering the environment to favor benign tumoral and peritumoral cells, instead of the traditional attacks on malignant cells, can be an effective adjuvant, or perhaps primary, anti-cancer treatment. Maley and colleagues [17] have also shown that ecological measures of clonal diversity predict progression to esophageal adenocarcinoma in Barrett's esophagus in a pattern that is in turn predicted by evolutionary oncology theory [6,10,17]. More detailed phylogenetic analysis of tumor cell lineages may shed light on tumor progression in individual patients [9]. Mathematical models of tumor evolutionary ecology suggest that if selection is dominated by competition among diverse cell lineages, one lineage may adopt a "cheater" strategy leading to a "hypertumor"--the cheater clone growing as a tumor on its parent tumor [8,18,19]. Coevolution among lineages may also produce cooperative cell types that act in some ways like tissues. A similar pattern may arise if malignant cell lineages diversify to exploit different resources or changing environments [10,18].

Despite these contributions, application of an evolutionary perspective to clinical oncology still suffers from a lack of maturity. In particular, the theory of tumor ecology--the theater in which evolution occurs--is critical, yet not well characterized for most tumor types [8-10]. One reason for this hole in our theory is that tumor ecologies feature unique elements not observed in traditional ecological systems. For example, in prostate cancer, ecological elements like the hormonal environment, redox state, and tissue inflammation have no obvious counterparts in an ecosystem as traditionally defined, but may play key roles in cancer pathology. Androgens, for example, mediate proliferation, apoptosis, oxidative stress, and perhaps inflammation in the prostate, and are therefore likely primary mediators of selective pressure in the evolution of prostate cancer.

This particular neoplasm is interesting for a number of reasons in addition to its unique ecological features. It accounts for 25% of new cancer diagnoses and 10% of cancer deaths in American men [20]. Most men develop prostate enlargement at some point in life, and autopsies show that, worldwide, 90% of men in their 10th decade of life have developed at least preclinical prostate cancer [21]. The aggressiveness of diagnosed cancers varies widely, but over 90% of prostate cancer cases are diagnosed at local or regional stages for which survival approaches 100% [20]. The natural history of this neoplasm is widely believed to span decades in most men [21]. This long preclinical phase suggests a typically slow evolutionary progression toward the malignant phenotype; how ecological factors drive this selective process is not known.

Androgens, the male sex hormones, have long been central to the study and treatment of prostate cancer. Androgens are essential survival factors for prostate secretory epithelial cells and act by binding with the androgen receptor (AR). Testosterone, the primary androgen in the serum, is converted to 5*α*-dihydrotestosterone (DHT) by the enzyme 5*α*-reductase in the prostate [22]. Testosterone and DHT both bind to AR, but DHT is more active, displaying greater binding affinity and stabilization of the AR complex [23]. Upon binding, androgen:AR complexes are phosphorylated, dimerize, and translocate to the nucleus, where they bind to the promoter regions of target genes [24] to modulate the transcriptional activity of at least several hundred target genes.

The importance of androgens is readily demonstrated by rat castration models. Following castration, over 70% of androgen sensitive cells undergo apoptosis [22], and the prostate epithelial mass decreases dramatically to only 7% of its original mass at 21 days [25]. Exogenous androgens induce prostate regrowth [23,26], but high levels of androgen alone do not generally induce the prostate to grow beyond its normal size; androgen induced proliferation is apparently regulated by the normal prostate cell count, although the mechanism for this is unclear [26].

Androgens are essential for normal prostate development, and deficiencies in 5*α*-reductase can severely impair prostate development [22]. Not only are androgens essential for prostate development, but changes in the androgen environment appear to mediate age-associated tissue remodeling. Serum testosterone declines with age in both the rat and man, leading to heterogeneous tissue remodeling in both animals. In the brown Norway rat (*Rattus norvegicus*), spontaneous hyperplasia associated with increased AR expression in the dorsal and lateral lobes is observed, while the ventral lobe may atrophy with age [27]. Men often experience benign prostatic hyperplasia (BPH), a disorder generally considered unrelated to prostate cancer, that is characterized by extensive hyperplasia of the stroma in the prostate transition zone [27,28]. Such changes in the microenvironment may alter the prostate cell turnover, count, genetic instability, and stress, and thus affect the selective environment that aging epithelial cells are exposed to. Most clinical prostate cancers are AR-dependent, and this observation has motivated androgen ablation therapy. Such therapy consists of chemical or surgical castration, which reduces serum testosterone by up to 95%, but reduces intraprostatic DHT levels by only 50% [22]. More complete androgen blockade can be achieved by supplementing castration with anti-androgens such as flutamide, nilutamide, and bicalutamide, and such therapy is referred to as maximal androgen blockade (MAB) [24]. However, the benefit to MAB over castration is uncertain, and a large meta-analysis suggested that any additional benefit to MAB is only slight [29]. Most men respond initially to androgen ablation, and often experience dramatic cancer regression. However, most cancers progress to a hormone refractory (HR) state even with near total androgen ablation, and while time to progression can vary greatly [24], recurrence occurs on average between 12 and 18 months following treatment [22]. Most cancers are more aggressive following HR recurrence, there are no effective treatments for such cancers, and average survival following progression does not exceed 15 months [24]. These cancers are often referred to as androgen independent, but most retain at least some dependence on the AR for survival.

Several major mechanisms for hormone refractory recurrence have been identified. Modification of AR signaling, in one form or another, is the dominant theme in HR cancer progression. In a minority of cancers, DNA-based alterations to the AR allow it to bind to non-canonical ligands such as estrogen and cortisone, and in some cases, clinical AR antagonists such as flutamide [30]. AR gene amplification occurs in perhaps 30% of recurring HR tumors [22]. However, such genetic alterations do not occur in the majority of HR cancers. Upregulation of 5*α*-reductase has also been identified as promoting recurrence [31], and overexpression of AR coactivators is associated with progression [22].

Several androgen independent pathways also exist. Upregulation of bcl-2, bcl-x, and mcl-1 protect against apoptosis independently of androgens, and expression of such proteins has been found to increase with cancer progression [32]. Many growth factors can activate signal transduction cascades that phosphorylate and activate the AR in a ligand-independent manner [22,30]. Tyrosine kinase receptor activation can induce survival and proliferation independently of the AR. The epidermal growth factor (EGF) family of proteins, and in particular HER2, promote androgen independent cancer growth [22], as can fibroblast growth factors [33] and insulin-like growth factor-1 (IGF-1) [22].

Upregulation of the AR protein is perhaps the single most important pathway by which cancers achieve androgen "independence". Chen *et al*. [30] found that in 7 prostate cancer xenograft models, increased androgen receptor expression was the only change consistently associated with HR cancer progression. Increased AR levels promoted growth in a ligand-dependent manner. Higher AR also altered cofactor recruitment to be biased towards coactivators, and it was even shown that high AR expression converted AR antagonists to weak agonists. Rapid HR cancer recurrence in a xenograft model by Rocchi *et al*. was always associated with increased AR expression [31].

Castration and androgen blockade in aggressive cancer is not the only setting in which androgens are manipulated clinically. 5*α*-reductase inhibitors are commonly used to treat both benign prostatic hyperplasia and alopecia (hair loss). Finasteride (Propecia, Proscar), the most common 5*α*-reductase inhibitor, is commonly used to treat BPH, and can reduce prostate volume and improve symptoms [28]. However, BPH in man is characterized by stromal, not epithelial, overgrowth [34] and is characterized by an elevated stroma:epithelium ratio [33,34]. Finasteride treatment targets the epithelium, with little if any effect seen on the stroma [34], and it dramatically increases the prostate stroma:epithelium ratio [28].

Because of their role in protecting against apoptosis and promoting proliferation and the (transient) efficacy of androgen ablation therapy, it has long been thought that high levels of androgens play a causal role in prostate cancer development. The fact that eunuchs and men with genetic deficiencies in 5*α*-reductase do not typically experience prostate cancer, along with the fact that androgen deprivation causes cancer regression have long been cited in support of this notion. But, as Raynaud recently pointed out [35], such scenarios have little if anything to do with cancer development under the normal physiologic androgen range. In support of the high androgen hypothesis, in several animal models androgens were capable of inducing cancer, and some clinical studies have suggested a link between high testosterone and cancer incidence [26,35].

In 1999, Prehn [26] proposed an alternate hypothesis: that low levels of androgen creates selective pressure for prostate cells that are less dependent upon androgen for growth. Declining levels of androgen could result in hyperplastic foci resistant to atrophy and susceptible to further neoplastic transformation. In indirect support of this hypothesis, a number of clinical studies have failed to support the notion that high androgen levels increase the risk of prostate cancer [35-38], and some data suggests that low serum testosterone is associated with aggressive, therapy-resistant tumors. In a prospective study including 17,049 men, high serum testosterone did not increase risk of prostate cancer and lowered the risk of aggressive tumors [39], and Sofikerim *et al*. recently found a significantly increased risk of cancer detection in men with low versus high serum testosterone [36]. Such data has led many authors to conclude that normal or high androgen promotes normal differentiation and function in epithelial cells, protecting against rather than promoting carcinogenesis [35,39]. Such results do not necessarily indicate a causal link between low androgen levels and carcinogenesis, and other authors have suggested that low testosterone reflects the poorer health of those experiencing aggressive cancers [40,41].

Although the role of androgens in predicting the incidence of prostate cancer has not been definitively settled, a broad literature dating from at least 1981 has consistently demonstrated poorer response to hormonal therapy in men with low pre-treatment serum testosterone [40,42-48]. Testicular atrophy is particularly strongly associated with aggressive cancers and a very poor response to therapy [49].

The effect of finasteride, which also modifies the intraprostatic androgen environment, on prostate cancer risk is unclear and controversial. The Prostate Cancer Prevention Trial (PCPT) randomly assigned men to receive daily finasteride (5 mg) or placebo for 7 years. Prostate biopsy was performed either for elevated adjusted serum PSA or abnormal digital rectal exam (DRE) findings. Moreover, all consenting participants were given an end-of-study biopsy. Finasteride use reduced overall prostate cancer incidence by 24%, but increased the risk of high-grade cancer over 7 years: 37% of cancers were high-grade in the treatment group versus 22% in the placebo group [50]. This result has sparked much debate over whether the increased incidence of high-grade tumors was a pathological artifact. Finasteride significantly reduced the prostate size in those treated, and reduced prostate size can increase the probability of cancer detection in biopsy samples: a lower overall prostate volume increases the probability that tumor tissue will be present in a biopsy core. Therefore, a detection bias could explain the increased rate of high-grade cancer. Lucia *et al*. [51] have argued that detection bias and changes in prostate histology induced by finasteride may account for the increased risk of high-grade tumors seen in the PCPT. However, a large study by Briganti *et al*. [52] where prostatectomy controlled for detection bias indicated that smaller prostates tend to harbor intrinsically more aggressive cancers. Freedland *et al*. [53] also found the weight of resected prostate specimens to be inversely associated with disease grade and risk of progression. These findings suggest that the increased risk of high-grade cancer seen in the PCPT may not have been an artifact. A recent review of RCTs concluded that 5*α*-reductase treatment reduces overall cancer incidence but may increase the risk of high-grade cancer [54]. This issue is particularly relevant, as a recently published clinical guideline stated that healthy men may benefit from a discussion of the risks and benefits of taking finasteride for primary chemoprevention of prostate cancer [55]. In light of these controversies, we focus upon understanding how different androgen environments promote carcinogenesis.

Several other investigators have used mathematical models to study androgens and prostate cancer. Jackson [56,57], and later Ideta *et al*. [58], used models to study selection for androgen independent strains following androgen ablation in aggressive prostate cancers. Potter *et al*. [59] also developed an exhaustive model of serum androgen dynamics and their effect on healthy prostate growth. Unlike Jackson, Ideta, and their colleagues, we focus not upon the evolution of prostate cancer in response to hormonal therapy, but rather upon the role androgens play in the evolutionary theater of early, pre-clinical prostate cancer.

In particular, we explore the selection process for pre-malignant phenotypes using a simple mathematical framework that considers prostate growth mediated by androgens at both the receptor kinetics level and tissue growth level; we also model multiple strains competing within a tissue. In this model, prostate growth is restricted by the homeostatic prostate size. That is, we assume that homeostatic mechanisms that prevent unbounded prostate growth are intact. Therefore, we consider only the earliest stage of molecular oncogenesis, where changes in gene expression influence proliferative or apoptotic activity, but before phenotypes allowing tissue invasion or metastasis have arisen. We model the chemical kinetics of intracellular androgen conversion from testosterone to DHT and the binding of these ligands to the AR. This model is used to inform a tissue-level model of the proliferation and death of prostate epithelial cells. We characterize the basic dynamics of both the AR kinetics and the coupled kinetics-growth model. We determine how both prostate epithelial mass and cell turnover rates change under different hormone environments.

Finally, this model is applied to an evolving, heterogenous cell population in which cell strains vary in AR expression. We focus upon the evolution of AR expression because of its deep importance in hormone therapy resistance and the fact that higher AR expression has been correlated with higher grade tumors [22,37]. We find that low serum testosterone strongly selects for greater AR expression. We also find that treatment with finasteride (i.e. 5*α*-reductase inhibition) similarly selects for increased AR expression. Together, these results suggest that low androgen environments select more strongly for hormone therapy resistant and possibly more aggressive cancer strains than do normal or elevated androgen environments.

## Methods

### Mathematical Model

We develop a minimal model of proliferation and apoptosis in prostate epithelium mediated by androgens. We model this on two levels: the first is a chemical kinetics scheme of the intracellular activity of androgens and their binding the AR. The second level of the model correlates the intracellular concentration of DHT:AR and T:AR complexes with proliferative and apoptotic activity.

Potter *et al*. [59] developed a thorough model describing androgenic regulation of prostate growth in the rat, and we are indebted to their model as a guide.

#### Chemical Kinetics Model

The intracellular chemical kinetics model is outlined as follows:

1. Free testosterone influx into the prostate is an empirical function of serum testosterone concentration, and this testosterone is uniformly distributed to the intracellular compartment of all prostate cells.

2. Free intracellular testosterone is converted to free DHT by the enzyme 5*α*-reductase. The intraprostatic 5*α*-reductase level is assumed to be a constant.

3. Free testosterone and DHT both degrade according to first-order kinetics.

4. Free testosterone and DHT bind to AR to form T:AR and DHT:AR complexes according to mass action kinetics. These complexes do not degrade.

5. Intracellular free AR binds to testosterone and DHT according to mass action kinetics, degrades by first order kinetics, and is produced at a rate that depends upon the homeostatic AR concentration and current free AR concentration.

The following variables are considered:

1. *T*_*S*_(*t*) = Total serum testosterone concentration (nM),

2. *R*(*t*) = Free intracellular androgen receptor concentration (nM),

3. *T *(*t*) = Free intracellular testosterone concentration (nM),

4. *D*(*t*) = Free intracellular DHT concentration (nM),

5. *C*_*T:R*_(*t*) = T:AR complex concentration (nM),

6. *C*_*D:R*_(*t*) = DHT:AR complex concentration (nM).

The basic mass action binding between T and DHT with AR and the conversion from T to DHT by 5*α*-reductase is illustrated schematically as

Translating this scheme into an ODE, and also taking into account T influx, AR production, and free T, DHT, and AR degradation yields the following chemical kinetics model:(1)(2)(3)(4)(5)

The rate of AR production is given as *λ*, and AR, T, and DHT degrade at rates *β*_*R*_, *β*_*T*_, and *β*_*D*_, respectively. 5*α*-reductase converts T to DHT by Michaelis-Menten enzyme kinetics, where *α *is the concentration of 5*α*-reductase, *k*_*cat *_is the turnover number, i.e. the maximum rate at which T is converted to DHT by each unit of enzyme, and *K*_*M *_is the Michaelis constant. Parameters  are the mass action rate constants for T and DHT binding to AR.

We have also assumed that all prostate androgens are intracellular and uniformly distributed among the epithelial cells. We ignore all the details of transport between serum, extracellular, and intracellular compartments, and instead have testosterone transported directly into the intracellular compartment. We leave U, the rate of testosterone influx into the intracellular prostate compartment, an unspecified function of the serum testosterone level,

The exact form for this function is determined empirically in the section on parametrization. Serum testosterone (*T*_*S*_), while in reality a function of time, is always imposed in our model and does not vary according to a governing ODE. Significantly, we have not modeled dimerization of androgen:AR complexes or their nuclear localization and binding to gene promoter regions under the assumption that the concentrations of androgen:AR complexes can be taken as surrogates for such activities.

We assume that prostate cells maintain a constant concentration of total AR (the sum of bound and unbound AR). We define *R*_*t *_as the homeostatic AR concentration, and at steady state we have:(7)

Moreover, we assume that total AR concentration is maintained at *R*_*t *_by modification of the AR production rate, *λ*, as follows:(8)

where  is the normal AR turnover rate. A more extensive discussion of this assumption is included in the AR kinetics parametrization section.

We also pay special attention to the effect of 5*α*-reductase inhibition by finasteride. Finasteride is a competitive inhibitor of 5*α*-reductase, implying that it increases the effective *K*_*M*_. The rate of conversion from T to DHT by 5*α*-reductase is modified to(9)

Where

*I *is the concentration of the inhibitor and *K*_*I *_is its dissociation constant.

#### Prostate Growth Model

We now link intracellular androgen concentrations to the proliferation and apoptosis of prostate epithelial cells. While we generally refer to low or high androgen levels causing a behavior, it is really the concentrations of AR:T and AR:DHT complexes that mediate these androgen related activities. We introduce the variable *C*_*t *_to represent the "effective" androgen:AR concentration. In [60], DHT was 2.4 times as potent as T in maintaining prostate weight and duct lumen mass, and these quantities varied linearly with the concentration of either androgen. Therefore, we take *C*_*t *_to be a simple linear combination of *C*_*T:R *_and *C*_*D:R*_,(10)

Thus, *C*_*t *_has units of nM and represents the concentration of AR:T equivalents. This approach allows the previously developed androgen kinetics model (equations 1-6) to be coupled directly to a model of prostate growth mediated by androgens. We let *P*(*t*) represent the number of prostate epithelial cells. We assume that the change in *P *is governed by two distinct death and proliferation signals; the per-capita proliferation rate is *M*(*C*_*t*_, *S*) and the per-capita death rate is *N*(*C*_*t*_, *S*), implying the basic model framework:(11)

where *S *represents oxidative stress. We now determine the formal forms for *M*(*C*_*t*_, *S*) and *N*(*C*_*t*_, *S*). Prostate epithelial proliferation and death are regulated by androgens in several ways:

1. Androgens induce the prostate stroma to produce factors, mainly bFGF and FGF-7, that support epithelial growth in a paracrine manner by supporting the prostate vasculature, inducing epithelial proliferation, protecting the epithelium from apoptosis, and regulating AR protein levels.

2. Androgens may have a direct mitogenic effect upon epithelial cells through upregulation of proteins required for cell cycle progression.

3. Androgens directly protect against apoptosis by negatively regulating TGF-*β *and increasing bcl-2 levels.

4. Androgens mediate oxidative stress and the production of reactive oxygen species (ROS) within epithelial cells which can induce proliferation, stasis, or death, depending upon the concentration.

In response to androgen withdrawal, rat prostate mass decreases dramatically through both apoptosis and atrophy. For modest androgen ablation, cellular atrophy and loss of prostatic fluid through loss of secretory function is primarily responsible for lost mass [60]. More extreme ablation, typically through castration, results in rapid and widespread epithelial cell apoptosis [22,25,61].

Androgens clearly protect against epithelial apoptosis. TGF-*β *expression by prostate epithelium is negatively regulated by androgen [61], and this factor appears to be necessary for apoptosis in response to low androgen [22]. bFGF produced by the stroma in response to androgens increases prostatic bcl-2 [22], an important anti-apoptosis protein that has also been implicated in late-stage prostate cancer progression [30]. Androgens also positively regulate the expression of a number of proteins that protect against apoptosis and downregulate others that induce apoptosis, such as TIMP3 [62].

It is generally accepted that androgens induce epithelial proliferation *in vivo *when the cell count is below normal, and androgen administration following castration induces rapid prostate regrowth in the rat [23,61]. However, proliferation is thought to be limited, at least to some degree, by the homeostatic size of the prostate [26,63]. It is unclear to what extent androgens are directly mitogenic for prostate epithelium, since much of the mitogenic effect is due to stroma-epithelium interactions mediated by androgens, and many *in vitro *studies have failed to demonstrate epithelial proliferation in response to androgen alone [64]. Several proteins required for cell cycle progression are positively regulated by androgens (cyclin D1, cdc2, PCNA), as are several that inhibit the cell cycle (geminin, GADD45*γ*) [62]. Androgens induce stromal production of bFGF and FGF-7, which have weak and strong mitogenic effects on the epithelium, respectively [33]. Interestingly, bFGF also reduces levels of AR in prostate epithelium, and while bFGF independently induces epithelial proliferation, it can slow androgen induced growth [65]. bFGF and vascular endothelial growth factor (VEGF) both are produced by the stroma in response to androgens and play an essential role in supporting the prostate vasculature [22].

Many studies focusing on the rat ventral prostate have suggested that high androgen levels alone can induce prostate epithelial cell hypertrophy but not hyperplasia [66]. Several studies by Banerjee *et al*. [27,67] on the brown Norway rat demonstrated that high androgen could induce hyperplasia in the lateral and dorsal prostate lobes, but not the ventral, and that this was related to increased AR expression in these lobes. Furthermore, aging was correlated with spontaneous hyperplasia in the dorsal and lateral lobes despite declining serum testosterone levels [27,66]. This hyperplasia was itself correlated with increased AR expression [27]. Saturation of the AR by its ligand therefore may be at least partially responsible for the failure of excess androgen to induce hyperplasia under normal conditions. This notion is supported by our model parametrization, where we estimate the normal intracellular concentrations of AR and DHT to be 45 nM and about 40-60 nM, respectively. An alteration in the testosterone:estrogen ratio in the aging rat was also suggested as having a possible causal connection to hyperplasia.

Thus, while normal prostate cell count is generally maintained, age-associated changes in the hormonal milieu and AR levels are likely able to induce hyperplasia and excessive androgen-induced proliferation. In our model, we assume that high levels of androgen directly induce proliferation while low levels cause apoptosis.

The prostate redox state is also influenced by androgens, and this may be deeply important in epithelial death, proliferation, and carcinogenesis. The mitochondria are the major source of reactive oxygen species (ROS). A significant amount of superoxide anion  is produced as a side-product of aerobic respiration, and  can generate a number powerful oxidants through further reactions [68]. Ubiquitous ROS include , H_2_O_2 _and the hydroxyl radical (HO•) [69].

Androgen blockade induces the production of ROS and subsequent oxidative stress. Both finasteride and flutamide (an anti-androgen) dramatically increased the expression of the pro-oxidant enzyme L-amino oxidase-1 (LaO1), which generates H_2_O_2_, in the rat ventral prostate [70]. Tam *et al*. [69] showed that rat castration results in dramatic upregulation of ROS generating NADPH oxidases (NOXs) and downregulation of ROS-detoxifying enzymes, including superoxides dismutase 2 (SOD2) and thioredoxin. Androgen re-administration restored antioxidant defenses, but only partially reduced NOX expression. Several other antioxidant proteins were not affected by castration, but were upregulated upon androgen re-administration. Pang *et al*. [71] also found that the expression of a number of antioxidant genes was downregulated following rat castration and suggested that this is a possible mechanism for castration-induced apoptosis.

Vascular regression caused by androgen withdrawal precedes epithelial apoptosis, causing hypoxia and a dramatic increase in hypoxia inducible factor-1*α *(HIF-1*α*) [72]. Hypoxia impairs aerobic respiration, increasing mitochondrial ROS production. Such mitochondrial ROS is required for stabilization of HIF-1*α *[73]. Thus, in addition to direct effects on redox related enzymes, a low androgen environment also increases ROS levels by inducing a hypoxic environment.

Androgen administration has also been shown to induce oxidative stress. Tam *et al*. [74] found that administration of testosterone with 17*β*-estradiol increased several NOXs, nitric oxide synthases (NOSs), and cyclooxygenase-2 (COX-2), resulting in oxidative and nitrosative stress in the lateral lobe of the Noble rat. Ripple *et al*. [75] found that physiologic levels of DHT induced ROS in the LNCaP carcinoma cell line, and ROS generation preceded DHT induced proliferation. Thus, a normal androgen environment likely promotes a balance between antioxidant and pro-oxidant activity [69], but both low and high androgen environments are pro-oxidant. Therefore, in our model we assume that both low and high levels of androgen induce the formation of reactive oxygen species.

At low levels, ROS act as important intracellular signalling molecules. A number of transcription factors, including NF-*κ*B and AP-1 are redox sensitive, and modest levels of ROS are mitogenic. Davies [76] reported that exposure of fibroblasts to 3-15 *μ*M of H_2_O_2 _resulted in a mitotic response, while levels an order of magnitude higher (250-400 *μ*M H_2_O_2_) induced permanent growth arrest. Very high levels of H_2_O_2 _caused either apoptosis (500-1000 *μ*M) or necrosis (5-10 mM).

We assume both low and high concentrations of *C*_*t *_induce ROS and that there is some background level of ROS independent of androgens. We choose *S *to represent ROS and formally take,(12)

Here, *μ *is the background ROS level, and ROS is induced by both low *C*_*t *_and high *C*_*t *_according to the first and second Hill functions, respectively. The half-maximal *C*_*t *_for ROS induction by low androgen is *θ*_1_, and *θ*_2 _is the half-maximal *C*_*t *_for high androgen induced ROS. We leave the Hill coefficients *n *and *m *nspecified at this point. We leave *S *a unit-less measure, but for future parametrization we could let *S *represent, for example, H_2_O_2 _equivalents with units *μ*M.

Broadly, we assume that prostate proliferation is induced by increasing concentrations of such complexes, and a high cell count inhibits proliferation. Low AR:ligand complex concentration induces apoptosis, and there is always some small baseline turnover rate (1-2% of cells turnover daily in the healthy prostate [22]). We assume that modest levels of ROS induce proliferation, while higher levels cause growth arrest and apoptosis. These assumptions lead to our formal choices for *M*(*C*_*t*_, *S*) and *N*(*C*_*t*_, *S*),(13)(14)

The maximum per-capita proliferation rate is *r*, and the maximum death rate is *δ *+ *δ*_0_. The specific formal forms for *M*(*C*_*t*_, *S*) and *N*(*C*_*t*_, *S*) are chosen as follows. First consider *M*(*C*_*t*_, *S*), the per-capital rate of proliferation. The proliferation signal due to direct stimulation by androgens should increase monotonically with *C*_*t*_, but given the biophysical limitations on the rate that cells can grow and divide, it must be bounded for large *C*_*t*_. The commonly used Hill function is a reasonable heuristic choice that satisfies these criteria. Since androgens have an array of mitotic effects we expect some cooperativity in the overall response and take the Hill coefficient to be 2, yielding a sigmoidal response curve. Any Hill coefficient greater than 1 would yield a sigmoidal curve--we use 2 as a first approximation. Thus we arrive at the function .

The proliferation signal due to ROS must pass through the origin, i.e. the response must be 0 when the ROS level is 0. It should be large for low *S *and, since growth arrest occurs at high ROS concentrations, it should attenuate as *S *becomes large. Qualitatively, the function *ϕS*exp(1 - *ϕS*) gives this behavior.

Finally, proliferation is attenuated as a nonlinear rate by the presence of other epithelial cells, and we use the term -*σP *to represent this growth inhibition. This second-order attenuation of growth is equivalent to a logistic model, and a possible alternative approach would be to attenuate *M*(*C*_*t*_, *S*) according to an explicit logistic term with a carrying capacity. This term prevents unbounded growth and causes prostate epithelial count to have an equilibrium point for any *C*_*t *_and *S*.

Now consider *N*(*C*_*t*_, *S*), the per-capital death rate. The death rate due to low androgen must increase monotonically as *C*_*t *_decreases. Since such death is generally due to the orderly process of apoptosis, it will occur at a finite maximum rate when *C*_*t *_= 0. Similar to the mitotic signal, the apoptotic signal can therefore be modeled by a Hill function: . The death rate due to high ROS levels should increase as *S *increases. While for very large *S *there may not be a well-defined maximum death rate as massive cellular insult will result in necrosis, it is unlikely that necrosis will occur under the physiologically feasible range for *S*. Therefore, we assume apoptosis occurs at a defined maximum rate for large *S *and again choose a Hill function to represent the apoptotic response to ROS: *S*^*q*^/(*ω*^*q *^+ *S*^*q*^). Apoptosis also occurs at the background per-capita rate of *δ*_0_:

Our incorporation of *S *as a function of *C*_*t *_into the equation for *dP*/*dt *allows both the direct and indirect ROS mediated effects of androgens on prostate growth to be incorporated into a single differential equation. This construction is somewhat similar to the ecological model of planktonic algae interaction with vegetation in shallow lakes by Scheffer *et al*. [77].

#### Prostate Evolution Model

Because of the importance of the AR in prostate cancer progression, we investigate how different androgen environments select for cell strains expressing different levels of the AR, i.e. we examine selection upon *R*_*t*_. To model competition between a large number of cell strains that evolve in time, we propose a state-transition model where cells transition between states that each represent a different level of *R*_*t *_expression. To derive the model, we make the following assumptions:

1. All epithelial cells express a constant amount of the AR (*R*_*t*_). Cells can mutate to express more or less AR^. ^Each mutation changes AR expression by only a small amount.

2. There are a finite number of prostate epithelial strains, each of which expresses a different level of the AR. A strain is defined by the level of AR expression. Therefore, mutation causes cells to transition between strains (or states).

3. All cells within each strain proliferate and die according to the prostate-growth model.

More formally, we define a set of states:(15)

Each state represents a strain of prostate cells that express a different total AR concentration (i.e. *R*_*t*_), and *R*_*t *_varies linearly with *i*. Cells transition from *P*_*i *_to *P*_*i*-1 _and *P*_*i*+1 _at first-order rate *γ*, representing mutation. The effective androgen concentration for strain *i*, , is determined according the chemical kinetics model, as is the ROS level, *S*_*i*_. Populations of each strain grow according to the prostate-growth model. The growth model is modified so that each strain grows independently, but growth is attenuated according to the total prostate cell count, Σ*P*_*i*_. Formally,(16)

For example, assume that there are 100 states (*Q *= 100) representing epithelial cell strains with *R*_*t *_ranging from 15 to 114 nM. The cells in, say, state 50, have *R*_*t *_= 64 nM. The cells in this state mutate out of the state at rate 2*γ*, representing even distribution to states 49 and 51. Moreover, cells from state 49 and from state 51 each mutate into state 50 at rate *γ*. In state 49, *R*_*t *_= 63 nM and in state 51, *R*_*t *_= 65 nM. Proliferation and death occurs in each state according to the growth model, as represented by the  terms.

This approach is designed to predict how quickly the average level of AR expression in the prostate will increase under different androgen environments, and to what level the AR will ultimately be expressed. The average level of AR expression for the prostate as a whole is calculated as a weighted average:(17)

### Parametrization

#### AR Kinetics Parametrization

Since we impose serum T (*T*_*S*_), we need to know the likely physiologic range of this variable. In brown Norway rats, serum T averaged 5.03 nM and 4.85 nM in young rats and 3.19 and 2.77 nM in old rats in [78] and [66]. Therefore, 3-6 nM is the likely normal physiologic range of serum T in rats.

We note that there are a number of published values for testosterone and DHT serum and prostate concentrations for both humans and rats. In [79], serum T was 4.95 nM, serum DHT was 1.3 nM, prostate T was 11.8 nM, and prostate DHT was 40.6 nM for the rat. Other authors have reported intraprostatic DHT concentrations 15-20 times T concentration [60]. In the rat, prostate T and DHT concentrations were 2.15 nM and 61.29 nM in [60] and 1.8 nM and 53 nM in [25]. In man, prostate T and DHT were 1.5 nM and 18.2 nM in [34]. Prostate testosterone concentration can be much higher than serum concentration [60,79], suggesting that most is intracellular. DHT prostate concentration can be over 50 times that of serum concentration, is produced in the intracellular compartment of the local tissue, and thus must be nearly entirely intracellular.

Values for most of the basic kinetic parameters , and *K*_*M *_are available directly from empirical biological data; these values with references are given in Table 1. The other parameters, *λ*, *U*, *α*, *β*_*D*_, have been estimated from a combination of empirical data and steady state analysis.

**Table 1:** AR Kinetics Parameters. Parameters and baseline values for the AR kinetics model.

Parameter	Meaning	Value	Reference
	T:AR rate of association	.14 nM ^-1^hr ^-1^	[80]
	T:AR dissociation rate	.069 hr^-1^	[80]
	DHT:AR rate of association	.053 nM^-1 ^hr ^-1^	[80]
	DHT:AR dissociation rate	.018 hr ^-1^	[80]
*β*_*R*_	Free AR degradation rate	ln(2)/3 hr ^-1^	[82]
*β*_*T*_	Free T degradation rate	ln(2)/3 hr ^-1^	
*β*_*D*_	Free DHT degradation rate	ln(2)/9 hr ^-1^	[61], see text
*α*	5*α*-reductase concentration	5.0 mg L ^-1^	see text
*k*_*cat*_	Rate of T → DHT conversion by 5*α*-R	18 ± 15 nmol hr ^-1 ^mg ^-1^	[97]
*K*_*M*_	Michaelis constant for 5*α*-R	75 ± 33 nM	[97]
*K*_*I*_	Michaelis constant for finasteride	.46 ± .21 nM	[97]

Several groups have measured binding kinetics for testosterone and DHT to the AR. Wilson and French [80] measured *k*_*a *_= .14 nM^-1 ^hr ^-1^, *k*_*d *_= .069 hr^-1^, and *K*_*D *_= .49 nM for testosterone, and *k*_*a *_= .053 nM^-1 ^hr^-1^, *k*_*d *_= .018 hr^-1^, and *K*_*D *_= .34 nM for DHT. In Dunning R-3327 rat prostate carcinoma, the *K*_*D *_of 0.5 nM for DHT was similar to normal tissues, but the dissociation half-lives for of 60 hr and 160 hr for testosterone and DHT, respectively, were much longer [81]. Nevertheless, testosterone always dissociates from the AR 2.5 to 3 times faster than DHT does [81].

The AR is unstable in the absence of its ligand. Gregory *et al*. [82] studied several androgen-dependent and recurrent cancer lines, and found that greater AR stability in the absence of ligand was associated with recurrence. Without ligand, the degradation half-life of AR was 3.0 hrs in androgen dependent LNCaP cells, and was greater than 12 hrs for recurrent CWR22 cells, a half-life reportedly comparable to that in the presence of ligand.

In the absence of any direct estimates, we use the following method to estimate the AR concentration within prostate cells. Wilson and French [80] reported a supernatant AR content in the rat prostate of 80-140 fmol/mg cytosol protein, and an AR content of 80-150 fmol/mg cytosol protein was reported for the rat Dunning R-3327 androgen-dependent prostate carcinoma [81]. A cDNA encoding the AR predicts a MW of 98,918 [83], and there are 200-300 g/L of protein in cellular interiors [84]. From this, it can be calculated that 1 fmol AR/mg cytosol protein corresponds to an intracellular concentration of 0.2-0.3 nM, and intracellular AR concentration is likely between 16 and 45 nM. This yields the likely total AR concentration, *R*_*t*_, which equals *R *+ *C*_*T:R *_+ *C*_*D:R*_.

**Figure 1:**
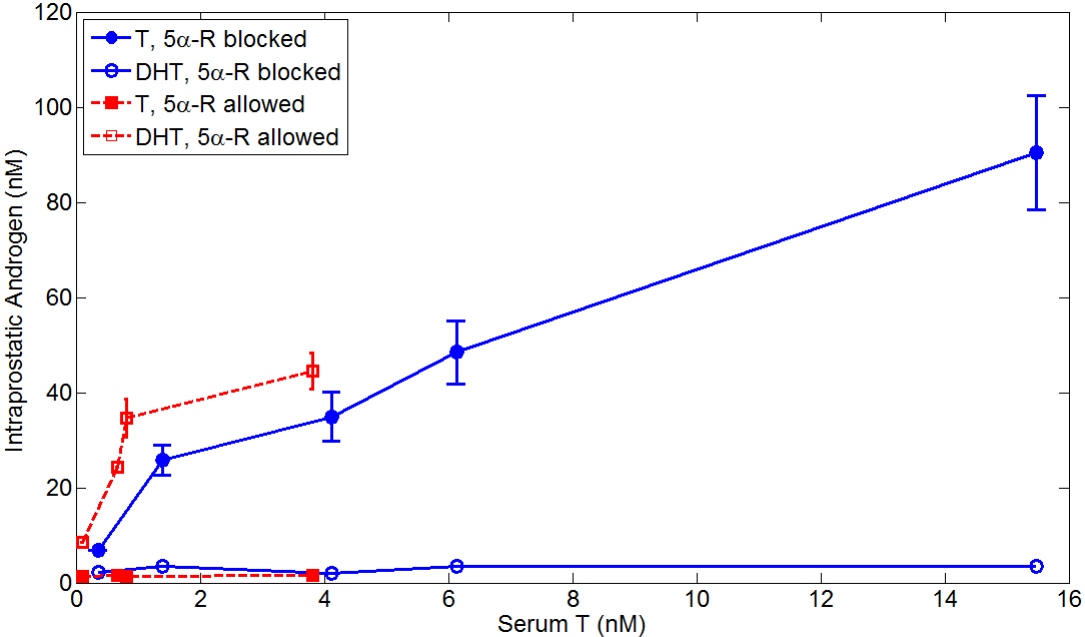
**Intraprostatic concentrations of T and DHT when 5*α*-reductase is and is not blocked (i.e. finasteride treatment) in the rat**. Data is from Wright *et al*. [60].

**Figure 2:**
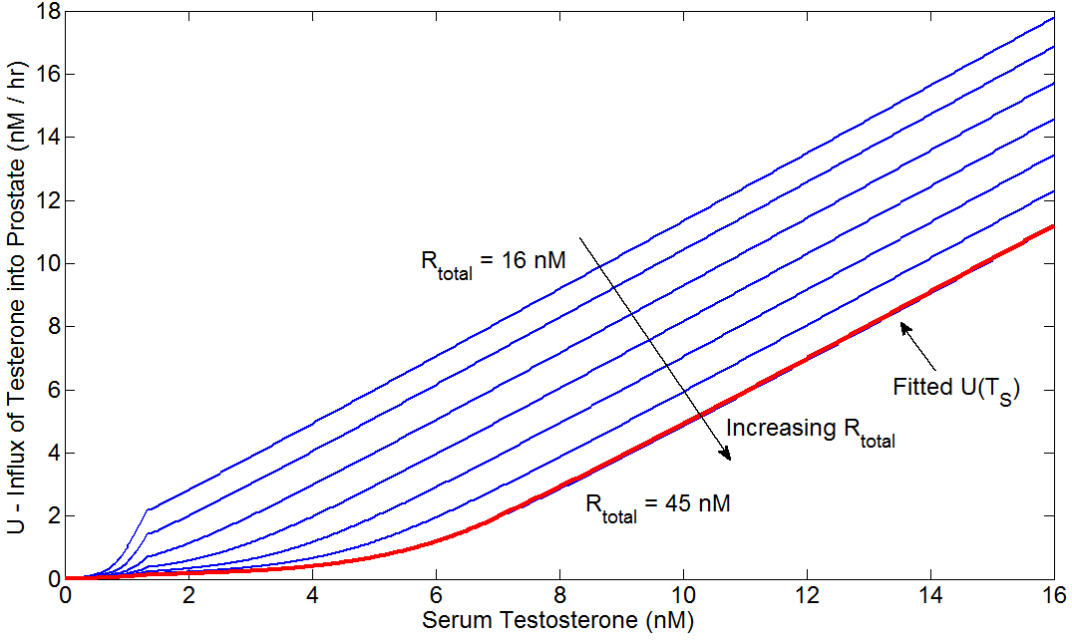
**Influx of free testosterone, *U*, as function of serum testosterone, as determined using the data of Wright *et al***. [60] The algebraic fit for *U*(*T*_*S*_) with *R*_*t *_= 45 nM is also indicated.

The influx of free testosterone into the prostate, *U*, is determined as an empirical function of serum testosterone based upon the data of Wright *et al*. [60], who measured intraprostatic testosterone and DHT concentrations as functions of serum testosterone in castrated rats both with and without treatment with the 5*α*-reductase inhibitor finasteride; this data is shown in Figure 1. We use the data-set where finasteride was present, and since almost no DHT was produced we simply take *α *= 0 (in reality, the effective *K*_*M *_has been made very large). We also ignore AR production and degradation and impose the total AR concentration *R*_*t *_= 16-45 nM, leaving *U *as the only free parameter. Running the system to steady state then allows *U *to be determined as a function of *T*_*S *_and *R*_*t*_, as shown in Figure 2. A higher *R*_*t *_implies a lower influx *U*, as when more AR is present, more androgen is bound and thus degrades more slowly. To determine *α *(5*α*-reductase concentration) and *β*_*D *_(DHT degradation rate), the data-set of Wright *et al*. where finasteride was not present is used in conjunction with the empirically determined function *U*. We have found that setting *R*_*t *_= 45 nM and using the corresponding function for *U *gives the best fit to the data. A good algebraic fit to the *U *curve for *R*_*t *_= 45 nM is(18)

**Figure 3:**
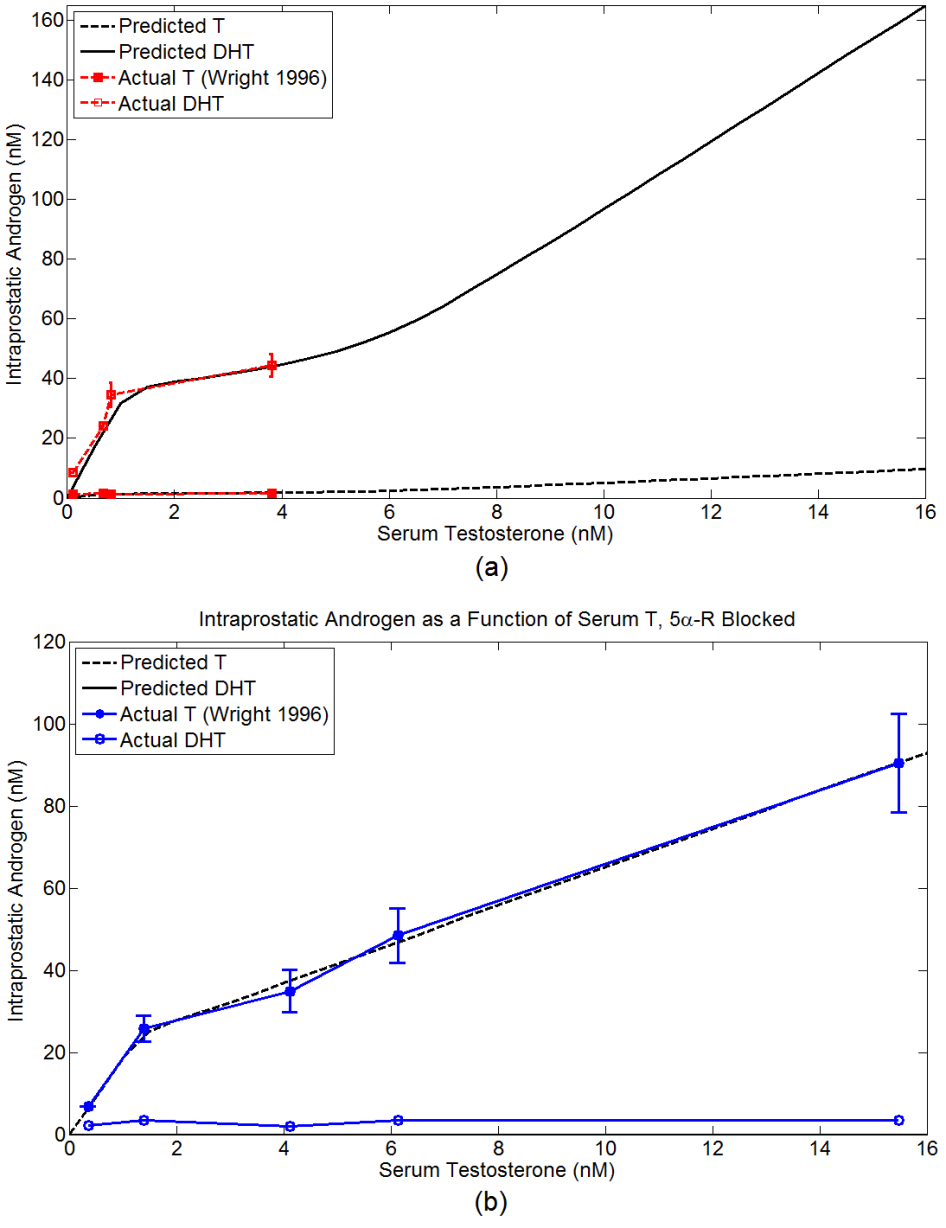
**T and DHT concentrations predicted by the AR kinetics model versus the actual concentrations in Wright *et al***. [60]**as a function of serum testosterone**. *R*_*t *_is fixed at 45 nM, all other parameters are fixed at the baseline values. (a) 5*α*-reductase allowed, *α *= 5 mg L^-1^. (b) 5*α*-reductase blocked, *α *= 0 mg L^-1^.

This is also indicated in Figure 1. From this data-set, we also estimate that *α *= 5 mg L^-1 ^and *β*_*D *_= ln(2)/9 hr^-1^. The half-life of DHT in the rat prostate is reportedly greater than 6 hours [61]; a degradation rate of *β*_*D *_= ln(2)/9 hr^-1 ^is consistent with this. Using these parameters, the predicted T and DHT concentration versus the actual concentration as a function of serum testosterone is shown in Figure 3. Predicted T and DHT concentrations for total 5*α*-reductase blockade (*α *= 0) versus actual values for finasteride treatment are also shown in Figure 3.

This parametrization shows that when the total AR concentration is a constant, a good fit to the data can be made, and suggests that AR production is regulated according to some homeostatic set point. We have been unable to favorably compare our results to the data of Wright *et al*. [60] if AR production, i.e. *λ*, is a constant. This implies that AR production may be positively regulated by free AR, or alternatively and more realistically, negatively regulated by androgen:AR complexes.

Indeed, AR mRNA and protein levels are clearly regulated by androgens, and this has mainly been studied in rat models of castration and androgen reintroduction. It has been consistently shown that AR mRNA levels increase dramatically following castration [27,85-87] and that androgens down-regulate AR mRNA in a receptor-mediated fashion [85]. However, castration also dramatically reduces AR protein levels [27,63,88]. Doorn *et al*. found that subsequent reintroduction of androgen rapidly increases the concentration of nuclear AR bound androgen, and the AR protein level rebounds to the pre-castration level within 2 days [63]. Steinsapir *et al*. found that cytosolic AR was replenished within 3 hours of testosterone reintroduction, and proposed that testosterone regulates AR levels through its effect upon receptor half-life [89].

Consistent with these results, Mora *et al*. [87] showed that, in previously castrated rats, administration of testosterone increased nuclear AR concentration after 1 hr. Testosterone administration for 1 hr did not affect mRNA levels, and positive regulation of transcriptional activity was ruled out as the underlying mechanism. Furthermore, inhibiting protein synthesis inhibited the increase in AR, and Mora *et al*. concluded that AR protein synthesis was involved in the mechanism for AR protein increase following testosterone administration.

Since AR binding to androgen greatly increases its stability, androgen administration could increase the level of AR protein by increasing its stability. These results are collectively consistent with the notion that mRNA production and AR protein synthesis are both negatively regulated by androgens, but that a nonlinear decrease in the stability of AR occurs for very low androgen concentration. We have examined the production rate, *λ*, and effective turnover rate of AR, , in our model when the total AR concentration is held constant. That is, we solve for *λ *using steady state analysis and use(19)

This gives  at steady state. We have found that AR is bound for much of the time for all but very low androgen concentrations, and that the effective turnover rate does indeed increase nonlinearly as serum testosterone goes to 0. This result may explain the contradictory results that variously imply positive or negative regulation of AR by androgens and the puzzling disconnect between increased AR mRNA levels but decreased protein levels seen after castration.

While such results imply that the AR level is not constant, castration is an extreme scenario (especially in the rat), and since AR levels rebound shortly after androgen reintroduction [63,89], it is likely that the AR level is actually relatively constant except in the case of extreme androgen deprivation. Therefore, we assume that there exists some homeostatic AR concentration, *R*_*t*_, that along with *C*_*T:R *_and *C*_*D:R *_determines the AR production rate(20)

where  is the normal AR turnover rate.

#### Growth Model Parametrization

The growth of the prostate epithelium, in our model, is governed solely by the intraprostatic weighted AR:ligand complex concentration, *C*_*t*_, and the prostate cell count, *P*(*t*). There are two independent proliferation signals, one mediated by *C*_*t *_and the other by *S*, and two similar death signals. In addition, the summed proliferation signal is attenuated by crowding and there is a background death rate. The shapes of these signals are collectively determined by the parameters *θ*_1_, *θ*_2_, *φ*_1_, *φ*_2_, *ϕ*, *ω*, *n*, *m*, and *q*. We have chosen values for these parameters based upon our determination of physiologic (and super- and sub-physiologic) parameter values for serum T and prostatic AR and the resulting *C*_*t*_s; they are reported in Table 2. We view these, therefore, as a first approximation.

**Table 2:** Growth Model Parameters. Parameters and baseline values for the prostate growth model.

Parameter	Meaning	Value
*θ*_1_	Half-maximal *C*_*t *_for low androgen induced ROS	30 nM
*θ*_2_	Half-maximal *C*_*t *_for high androgen induced ROS	225 nM
*μ*	Background ROS level	.05
*n*	Hill coefficient for low androgen induced ROS	4
*m*	Hill coefficient for high androgen induced ROS	4
*φ*_1_	Half maximal *C*_*t *_for high androgen induced proliferation	110 nM
*φ*_2_	Half maximal *C*_*t *_for low androgen induced apoptosis	40 nM
*ϕ*	Determines form of ROS induced proliferation signal	4
*ω*	Half-maximal *S *for ROS induced death	1
*q*	Hill coefficient for ROS induced death	8
*σ*	Mass action coefficient for proliferation attenuation	1.5 × 10^-10 ^cell^-1 ^hour^-1^
*δ*	Background death rate	.004 hour^-1^
*r*	Maximum per capita growth rate	ln(2)/24 hour^-1^
*δ*	Maximum per capita death rate	ln(2)/24 hour^-1^

**Figure 4:**
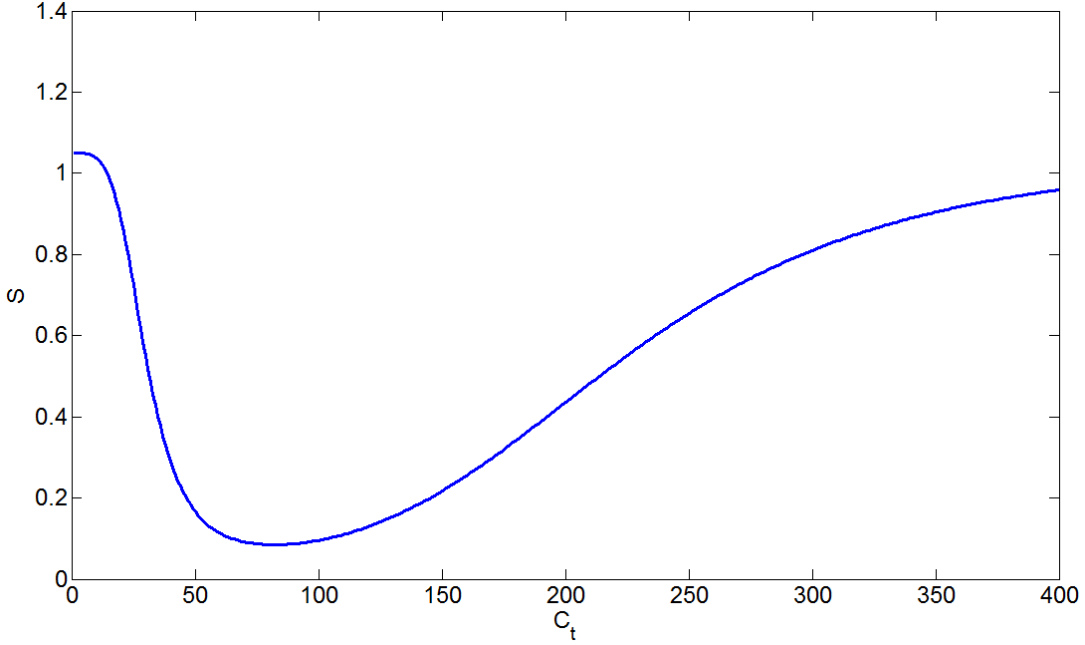
**Oxidative stress, *S*, as a function of *C*_*t*_**.

**Figure 5:**
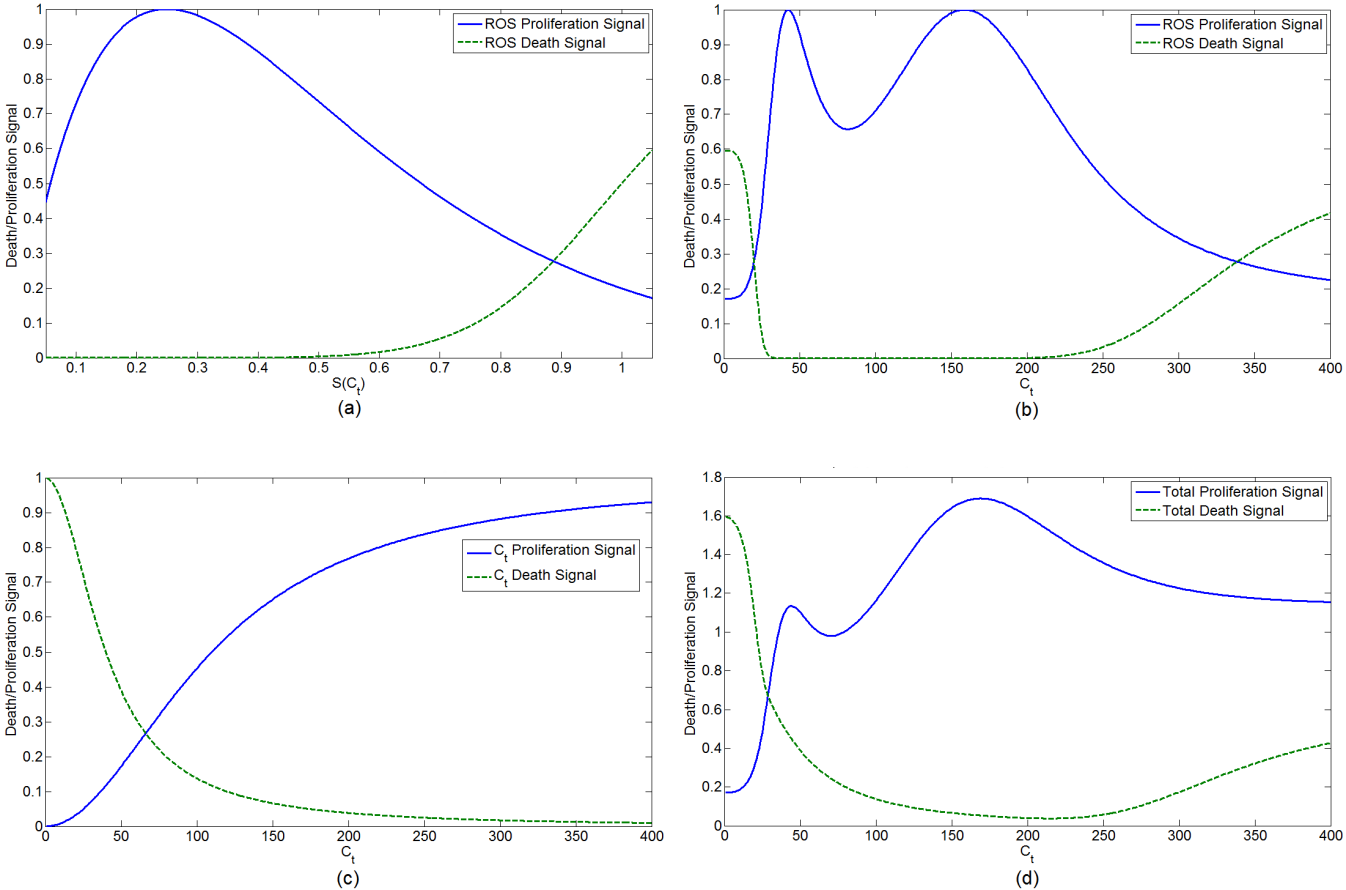
**Curves for the different growth signals**. Because the ROS level *S *is a function of *C*_*t*_, the proliferation and death signals mediated by *S *can be given as a function of either *S *(as in (a)) or of *C*_*t *_(as in (b)). Attenuation of proliferation by crowding (the -*σP*) is disregarded here because it affects the proliferation signal in a way that depends on the instantaneous prostate mass. (a) Prostate cell growth and death signals due to ROS as a function of *S*. (b) Prostate cell growth and death signals due to ROS given as a function of *C*_*t*_, as *S *is itself a function of *C*_*t*_. (c) Proliferation and death signal due to the effective AR:ligand concentration, *C*_*t*_. (d) Overall proliferation and death signals mediated by both ROS and AR:ligand concentration. Because ROS level is a function of the AR:ligand concentration, *C*_*t*_, the overall signal can be given as a function of only *C*_*t*_.

Oxidative stress, *S*, is shown as a function of *C*_*t *_in Figure 4. Figure 5 shows the proliferation and death signals directly due to *C*_*t *_and *S*. It also shows the proliferation and death signals mediated by *S *as a function of *C*_*t*_, since *S *is itself a function of *C*_*t*_. Such curves emphasize that *C*_*t *_is the ultimate mediator of all proliferation and death (besides crowding effects) in our model. These figures disregard the -*σP *crowding term as this varies depending upon prostate size. The qualitative form of these curves is preserved over most parameter space.

## Results and Discussion

### Basic Dynamics of the AR Kinetics Model

**Figure 6:**
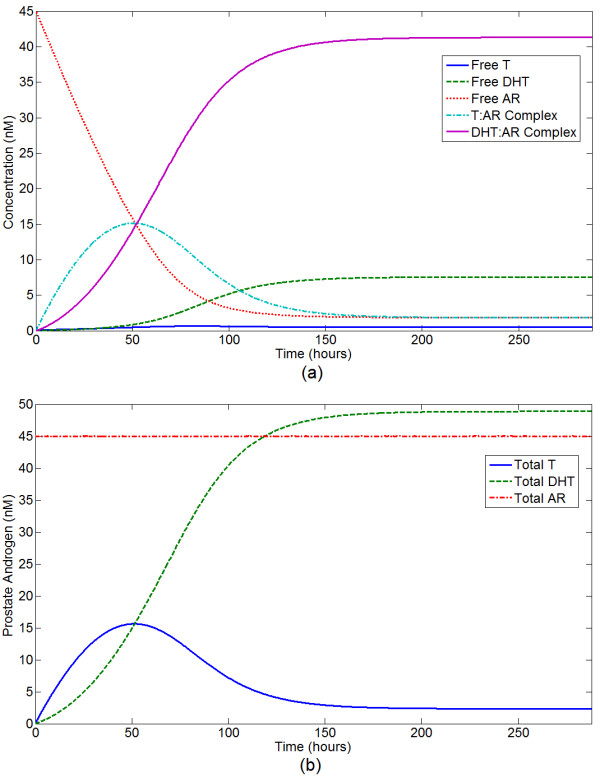
**Time-series for the AR kinetics model**. Free AR is set to 45 nM as an initial condition; all other variables are initially zero and baseline parameter values are used. Serum T is prescribed at 5 nM, inducing an influx of testosterone, and the model runs to a steady state. (a) Time-series for all model variables - free T, free DHT, T:AR complex, DHT:AR complex, and free AR. (b) Time-series for total T, DHT, and AR concentrations. (a) Time-series for all model variables - free T, free DHT, T:AR complex, DHT:AR complex, and free AR. (b) Time-series for total T, DHT, and AR concentrations.

We briefly characterize the basic dynamics of the AR kinetics model. The time-dependent dynamics of the model are demonstrated by initially setting *R*_*t *_to a constant and all other variables to 0. Serum T concentration is prescribed, and the model is run to steady state, as shown in Figure 6. For baseline parameter values, the free T concentration is always small, as most T is rapidly converted to DHT. There is a transient peak in T:AR complex concentration early in time, but the DHT:AR complex dominates within several days; this pattern is a consequence of the time it takes 5*α*-reductase to produce DHT. For physiologic values of serum T and prostate AR, once steady state is reached most androgen is bound its receptor, and nearly all intraprostatic androgen is DHT. These dynamics are biologically expected.

**Figure 7:**
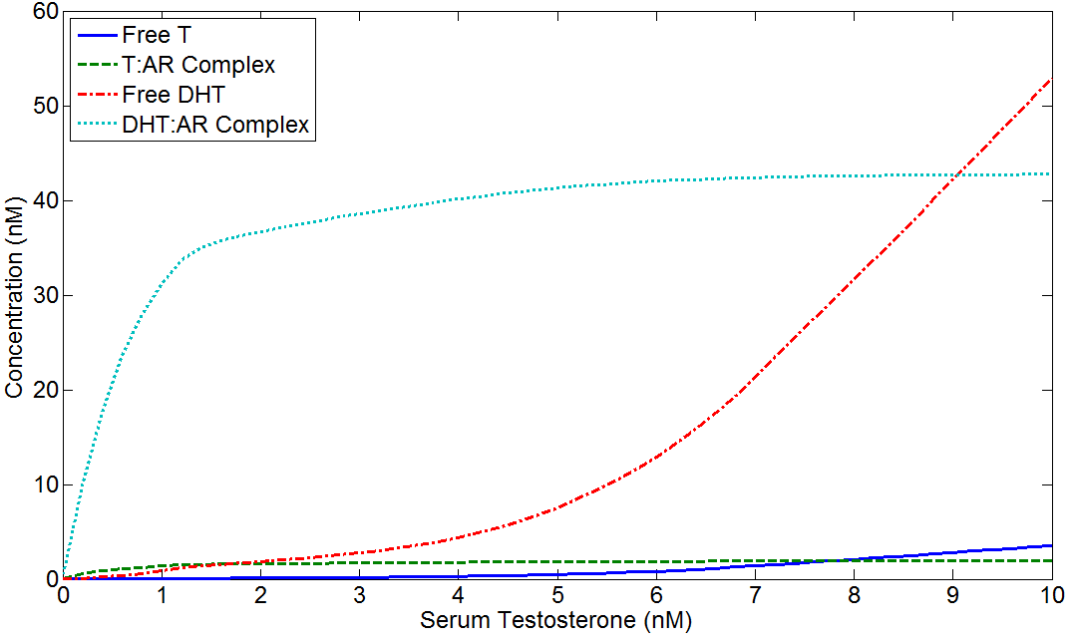
**Concentrations of free T, free DHT, T:AR complex, and DHT:AR complex at steady state as a function of serum testosterone**. *R*_*t *_is fixed at 45 nM.

At steady state, most AR is bound at physiological levels of serum testosterone. For superphysiological serum testosterone, intraprostatic free DHT increases while AR:ligand complex concentration levels off; intraprostatic free T also increases modestly with high serum T. This qualitative pattern is observed regardless of the concentration of AR; an example is shown in Figure 7. While this qualitative pattern is consistent, the absolute levels of bound androgens increase linearly with AR concentration regardless of serum testosterone.

### Sensitivity of AR Kinetic Parameters

We examine how altering several central parameters in the kinetics model influences the steady state AR:ligand concentrations under different androgen environments. Lea and French [81] found that while the *K*_*D *_for DHT:AR binding was the same in carcinomas as in normal tissue, the actual on/off rates were much slower. In our model, changes in the on/off rates do not affect the steady state as long as the *K*_*D*_s remain constant, suggesting slower rates offer no benefit to carcinoma growth. However, our model does not take into account the effect of increased complex life-spans, and it may be useful to consider average life-span in future work.

We examine the parameters associated with 5*α*-reductase: *α*, *k*_*cat*_, and *K*_*M *_and *η *(i.e. effective *K*_*M*_). All affect the steady state in a similar manner and change the ratio of DHT:total androgen in a nonlinear manner. The total amount of androgen in the prostate also decreases modestly in a nonlinear fashion when 5*α*-reductase is inhibited.

Finally, we investigate the degradation rates of *R*, *T*, and *D*. We find that for normal physiologic serum T, only *β*_*D*_, the degradation rate of DHT, has any significant effect on either total prostate androgen or the ratio of DHT:T; both decrease dramatically with increasing *β*_*D*_. At low serum T, *β*_*R*_, the degradation rate of free AR, is also important. Increasing *β*_*R *_significantly reduces total androgen, but does not affect the DHT:T ratio. Modifying the rate at which free T degrades, *β*_*T*_, has only minor effects.

Gregory *et al*. [82] found that increased AR stability in the absence of ligand was associated with recurrence of androgen-dependent cancer cell lines. Our model supports the notion that in a low androgen environment, increasing the stability of free AR (i.e. reducing *β*_*R*_) will augment the response to androgens by increasing the concentration of receptor bound androgen. Therefore, increasing the half-life of the free AR is one pathway through which cancers may overcome androgen blockade.

### Steady State Cell Count, Turnover

**Figure 8:**
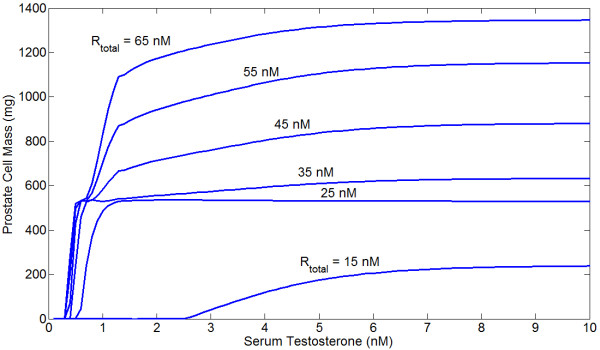
**Steady state prostate mass as a function of serum testosterone for different levels of *R*_*t*_**. Note that below *R*_*t *_= 25 nM mass quickly decreases for all serum T, while there is a reasonable stable region between 25 and 45 nM, after which mass increases roughly linearly with *R*_*t *_before leveling off. For higher values of *R*_*t *_than those displayed, increases in serum T can cause a reduction in prostate mass (see Figure 9).

**Figure 9:**
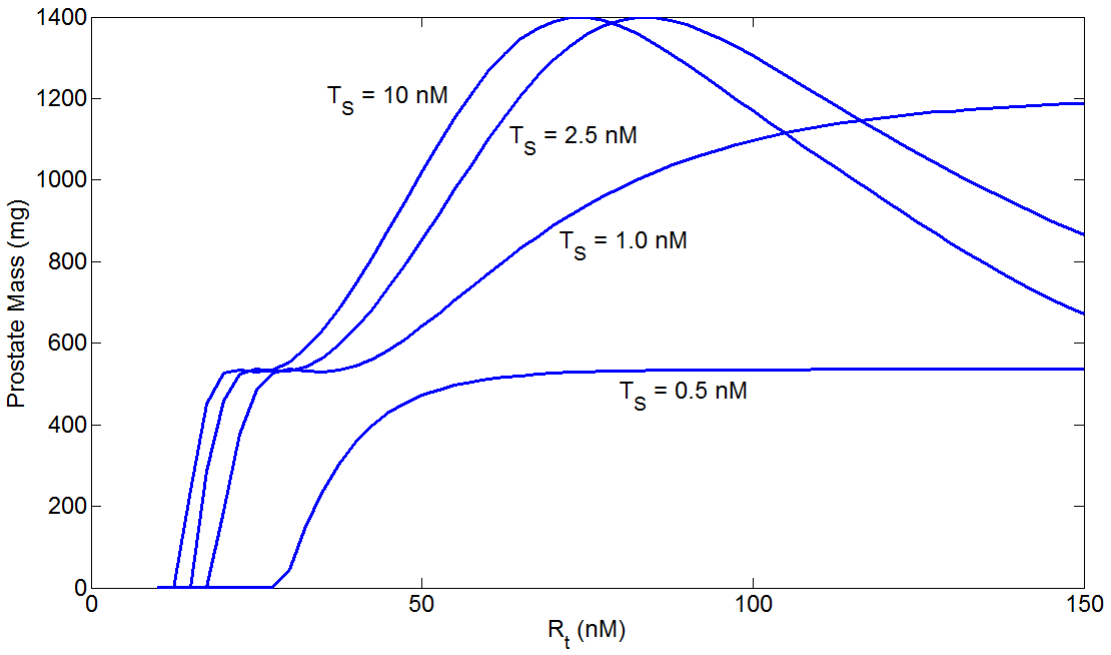
**Steady state prostate mass as a function of *R*_*t *_for different serum testosterone concentrations**. Note that for sufficiently high AR concentrations, increases in serum T actually reduce prostate mass.

We now examine the model dynamics at the prostate growth level. The steady state prostate mass as a function of serum testosterone for different levels of *R*_*t *_is shown in Figure 8, and mass as a function of *R*_*t *_for different serum T levels is given in Figure 9. Units have been converted from cell count to approximate mass, but due to the ad hoc parametrization of the growth model the absolute numbers are unimportant - it is the shapes of the curves that matter. We emphasize that we are not attempting to relate these curves to any specific data, but instead are exploring the qualitative behavior that results in a model derived from biological principles.

**Figure 10:**
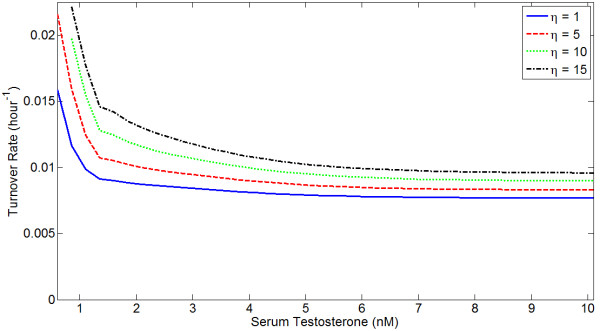
**Turnover as a function of serum T for different *η*s**. Higher *η *indicates greater 5*α*-reductase inhibition, and turnover increases with increasing *η*. Cell turnover decreases as serum T increases.

For higher levels of serum testosterone, prostate mass peaks at some level of *R*_*t *_and then declines. This decline occurs because the growth signal induced directly by androgens saturates for high *R*_*t *_(or *C*_*t*_) while ROS levels increase. This causes the ROS proliferation signal to attenuate, while the death signal increases in magnitude. While at steady state *dP*/*dt *= 0, i.e. there is no net growth or death, the internal cell turnover rate varies with *R*_*t *_and *T*_*S*_. This will affect the rate at which evolution can occur, as greater cell turnover will presumably increase the rate at which mutations accumulate. Lower androgen always increases the turnover rate in this model. Figure 10 shows turnover as a function of serum T for several levels of 5*α*-reductase inhibition.

We have performed a sensitivity analysis of all the growth model parameters and found that the qualitative shapes of the mass and turnover curves are preserved.

### Evolution and Selection for Elevated AR Expression

**Figure 11:**
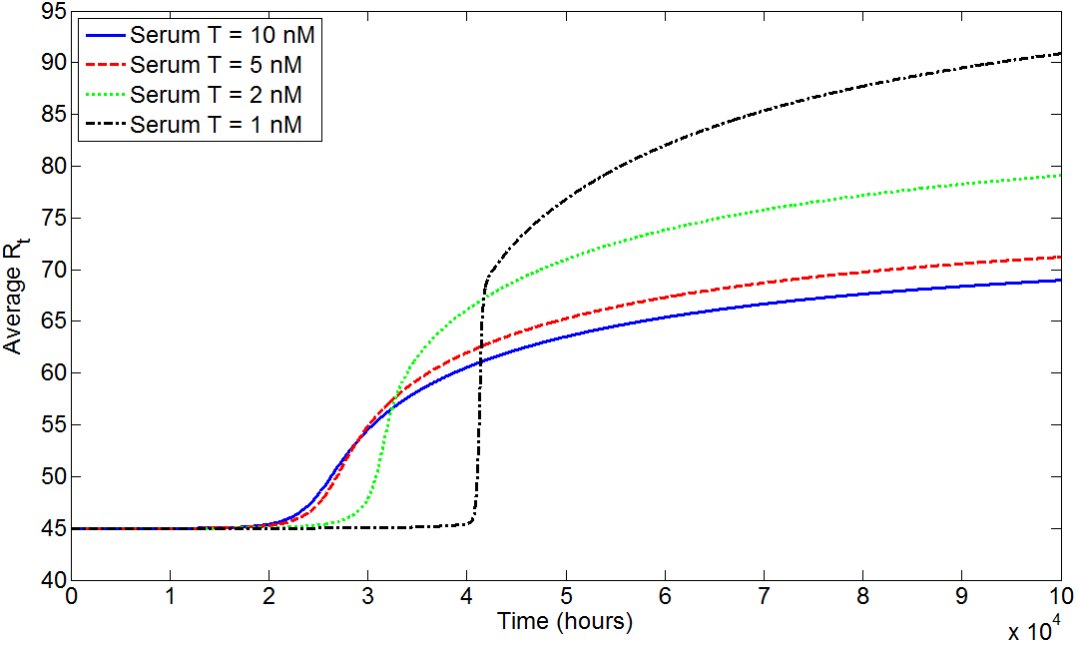
**Evolution of average *R*_*t *_in the state-transition model under different serum T**. AR expression is presumably a marker for the malignant potential of a strain. Low serum T selects for higher *R*_*t *_than the normal environment (serum T = 5 nM), but takes longer to do so. High serum T selects for a slightly lower *R*_*t*_.

**Figure 12:**
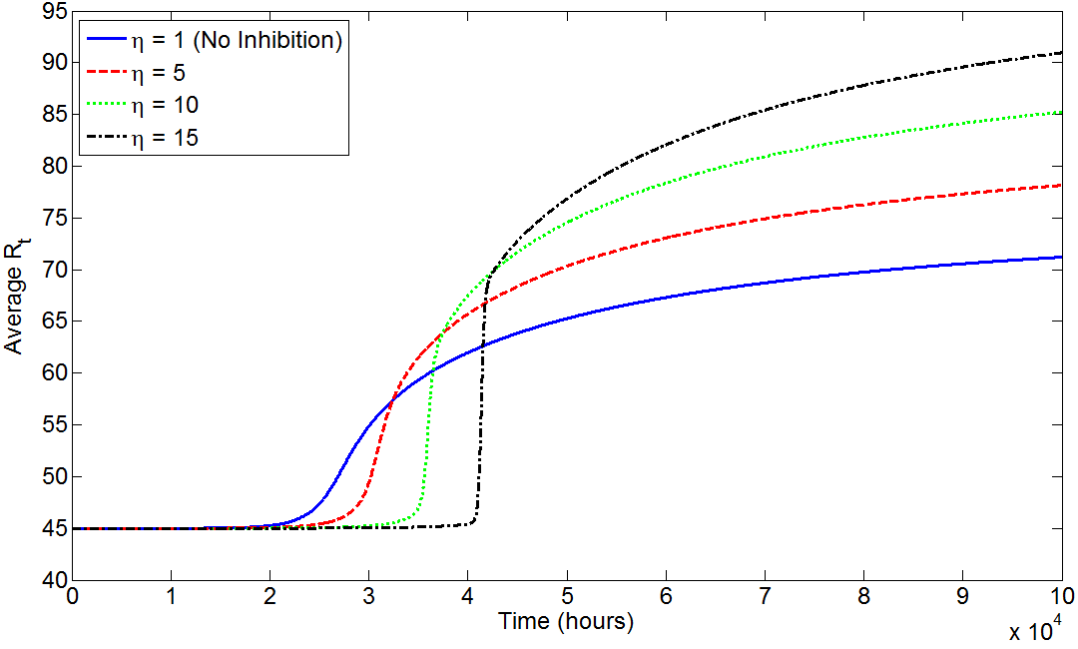
**Evolution of average *R*_*t *_in the state-transition model under different levels of 5*α*-reductase inhibition by finasteride**.

We use the state-transition model for the evolution of AR expression to determine how different androgen environments influence the prostate-wide evolution of AR expression. We choose our model to have 100 states representing *R*_*t *_from 15 to 114 nM. That is, we set *Q *= 100 and , the total AR level at state *i*, to be 14 + *i *nM, *i *∈ {1,2,...,100}. The total number of cells in each state is tracked through time, and the average *R*_*t *_at all time steps is calculated. Figure 11 shows the evolution of average *R*_*t *_under different serum T levels. 5*α*-reductase inhibition, e.g., through finasteride treatment, also induces a low androgen environment, and Figure 12 shows how evolution changes under increasing values of *η *(increasing effective *K*_*M*_).

In general, our results indicate that physiologic serum T selects for modestly increased AR expression, as do all androgen environments. Therefore, the model predicts that even in healthy men, prostate epithelial cells will increase their AR expression and hence potential for malignancy with time. Under high serum T, selection for an elevated *R*_*t *_is actually slighter weaker. In comparison, low androgen environments (i.e. low serum T or low 5*α*-reductase) demonstrate the greatest selection for increased AR expression. Generally, the widespread appearance of strains expressing high levels of AR occurs abruptly and later in time than under normal androgen levels. While this selection occurs later in time, a higher average *R*_*t *_is ultimately obtained.

**Figure 13:**
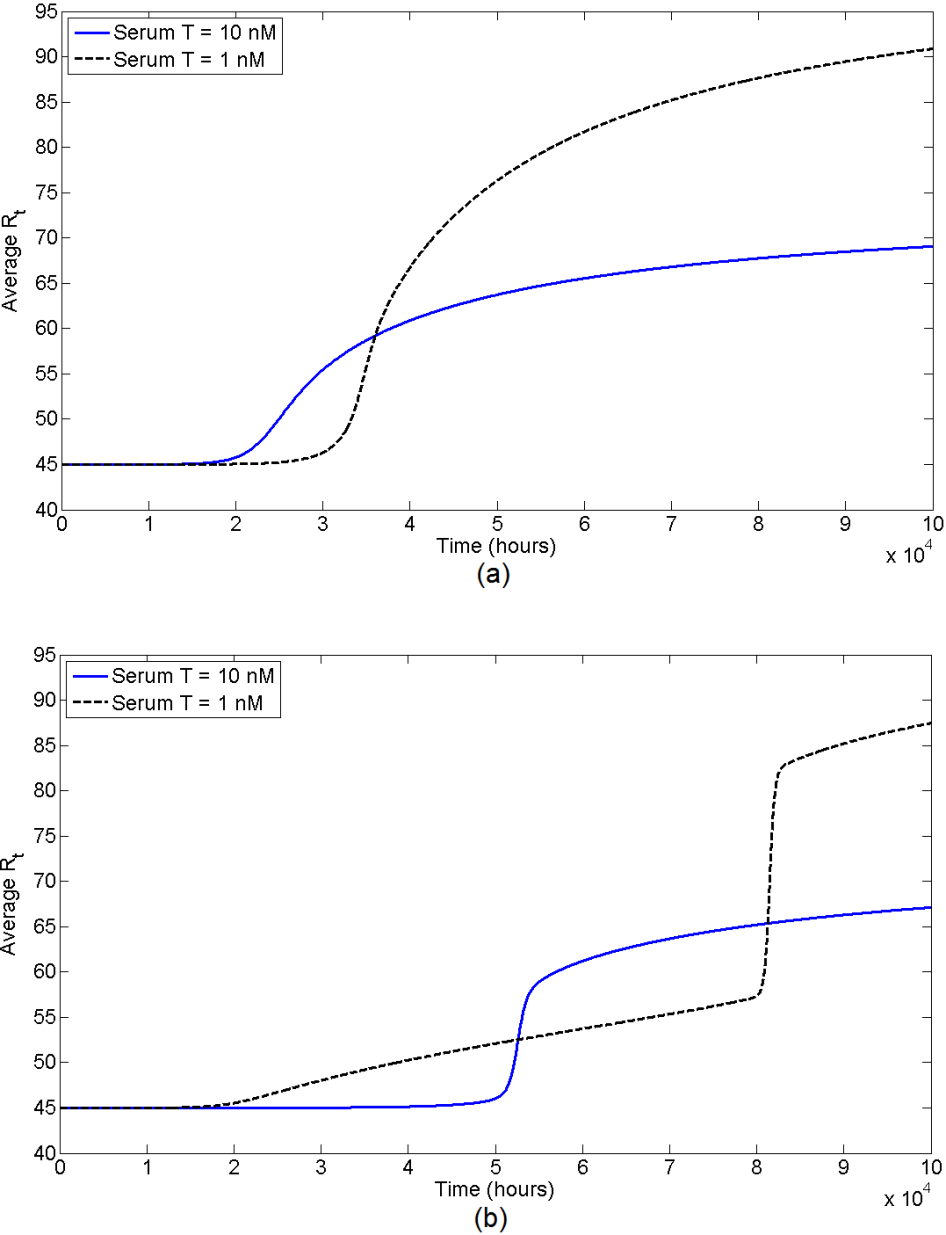
**Evolution of average *R*_*t *_in the state transition model under high and low serum testosterone concentrations for 2 different values of the *θ*_1 _parameter**. For *θ*_1 _= 20, the more typical pattern is observed with the appearance of AR overexpression occurring later in time but to a greater magnitude under the low androgen environment. A more unique pattern is seen for *θ*_1 _= 60. (a) Evolution of average *R*_*t *_for *θ*_1 _= 20. (b) Evolution of average *R*_*t *_for *θ*_1 _= 60.

We have performed a sensitivity analysis to determine whether this behavior is preserved under different growth model parameter values. We have found that the prediction that low androgen selects for a greater final *R*_*t *_is always preserved. The parameters *θ*_1_, *θ*_2_, and *μ*, which govern the level of ROS, have the greatest effect on the dynamics. Increasing *μ*, the background ROS level, causes low androgen to select for higher *R*_*t *_earlier in time. Decreasing *θ*_2_, which implies higher ROS for relatively lower levels of *C*_*t*_, also decreases the time to selection for *R*_*t*_. Increasing *θ*_1_, which implies ROS is elevated under a lesser degree of androgen insufficiency, changes the dynamics most significantly. Large values of *θ*_1 _result in a two-phase pattern of selection for high *R*_*t *_in low androgen environments: AR initially increases slowly, there is a sudden jump, and finally increases slowly again. This and a more typical pattern of AR evolution for low versus high androgen environments are shown in Figure 13. Overall, low androgen appears to select for a greater final *R*_*t *_in all parameter space, and this selection becomes apparent later in time than under normal androgen for most parameter space.

These results suggest that a low androgen environment may delay the development of a malignant phenotype, but result in a more malignant or therapy-resistant strain later in time. This result could also be interpreted to mean that low androgen reduces the overall incidence of cancer, as the expected time to the development of a malignant strain is increased, but those cancers that do arise may be more aggressive. This notion is consistent with the results of finasteride treatment in men [50].

## Conclusions

We have developed a preliminary model of the binding kinetics of prostate androgens, their relationship to proliferation and death in the prostate epithelium, and the evolution of AR expression, and hence malignant potential, in different androgen environments. Thus, we have modeled prostate carcinogenesis at three distinct scales. At the receptor kinetics scale, we have described the essential interactions between T, DHT, and AR, and have fully parameterized this model using biological data. At the prostate organ level, we have taken both the direct and indirect ROS mediated effects of androgens on epithelial proliferation and apoptosis into account. At the highest scale, we have employed a state-transition model to study selection between epithelial strains expressing different levels of AR, and whose behavior is determined by the coupled AR kinetics and prostate growth model. Results using this evolutionary modeling framework have suggested that a low androgen environment caused either by low serum testosterone or clinical intervention with 5*α*-reductase inhibitors can increase selection for AR overexpression in prostate epithelium, while high androgens may weakly protect against AR overexpression. As a whole, the clinical literature and our theoretical results suggest the following hypothesis: that low intraprostatic AR:ligand concentrations, caused either by low serum testosterone or 5-*α *reductase inhibition, drive evolution towards decreased dependence upon androgens in prostate epithelium, therefore increasing the risk of cancer that is resistant to hormonal therapy. Moreover, such cancers may arise later in life than those cancers with lower sensitivity to androgens.

We must emphasize the preliminary nature of the model, and there are likely many ways to model the phenomenon that we are studying. We have used only one approach, and there is much room for improvement as well as future work. In particular, we have not (explicitly) modeled the essential interactions between stromal and epithelial cells that mediate prostate growth. Moreover, we have ignored the heterogenous architecture of the prostate. Not only do different prostate lobes display different responses to androgens [66,67], but prostate epithelial cells are exposed to different levels of androgen and are differentially sensitive to such hormones.

We have not explicitly modeled the role of the prostate vasculature. Delivery of testosterone from the serum to the prostate is dependent upon prostate size and the state of the vasculature, both of which are themselves regulated by prostatic androgen, and vascular regression precedes prostate involution in castration models [22,72]. We have taken this into account to some degree through the use of an empirical function to describe testosterone flux into the prostate as a function of serum testosterone, as prostate weight is also a function of serum testosterone. However, we have assumed homogenous delivery to all cells, and considering the spatial dynamics of testosterone delivery is an important potential modification to the model. Moreover, vascular regression causes hypoxia and induces oxidative stress, which we have also partially taken into account by making *S *increase with low androgen, but hypoxia is also likely spatially heterogeneous.

While much mechanistic and spatial complexity has been ignored, the relative simplicity of our model of tissue-level prostate growth is also a strength in that it allows several of the competing effects of androgens on prostate epithelium--i.e., oxidative stress, proliferation, apoptosis--to be subsumed into a single differential equation. Thus, the model includes the essential effects of androgens with minimum complexity, and the qualitative effects of androgens on the prostate epithelium can be explored in a minimal formal setting. However, we have not rigorously parametrized this level of the model, although its qualitative behavior is preserved over a wide range of parameter values. It follows that a weakness of the model framework, in its current form, is the difficulty in confronting the model with quantitative data other than in regards to its qualitative predictions.

Our parametrization and investigation of the AR kinetics model has yielded some insight into AR dynamics. Our parametrization suggests that AR levels are constant under the physiologic range of serum androgens. We have found that increased stability of free AR has little effect on AR dynamics under normal androgen levels, but in a state of androgen deprivation, increased stability of the free receptor significantly increases intraprostatic AR:ligand concentrations, implying a role for such a modification in HR cancer, as seen in [82]. Moreover, decreasing 5*α*-reductase activity (through either *α*, *k*_*cat*_, or *K*_*M*_), can decrease AR:ligand concentrations in a nonlinear manner.

In our model, when serum T is low or 5*α*-reductase is inhibited, the prostate epithelial cell mass is much lower. Thus, there is simply less substrate for selection to act upon when serum T is low. However, in such an environment the cell turnover rate is higher. Therefore, mutations may arise more rapidly and be selected for more quickly in a low androgen environment despite the lower cell count.

This, along with the results from our state-transition model, support the notion that low androgen levels can induce selection for phenotypes with increased AR expression. Such a phenotype may be considered to have a higher potential for malignancy or be "pre-malignant." In particular, such a phenotype is likely to be resistant to androgen ablation therapy, and a great deal of experimental and clinical literature supports the notion that low androgen levels induce treatment resistant cancers. Several studies by Labrie *et al*. [90,91] on mouse mammary carcinoma cells cultured in different concentrations of DHT demonstrated that a single tumor line can yield wide phenotypic variety in sensitivity to DHT (*K*_*M *_for growth varied up to 1,250-fold in [90]) and that low DHT concentration selected for clones that were hypersensitive to androgen, requiring minuscule DHT levels for growth. Such hypersensitive clones would likely demonstrate little if any response to androgen blockade.

A number of clinical studies have shown that low testosterone levels predict a poor response to androgen deprivation. In 1981, Adlercreutz *et al*. [43] found that men with lower serum testosterone had a poorer response to androgen deprivation. A number studies over the following decade consistently found low serum testosterone to be associated with poor responses and shorter survival [40,42,44,45,48]. In one study of metastatic cancer, pretreatment serum T was a stronger predictor of treatment response than the extent of bone metastases [46,47]. Furthermore, in some cases high serum T predicted a better response to therapy [43,48].

Daniell [49] reported men with testicular atrophy presented with highly undifferentiated tumors and had a much worse prognosis following orchiectomy than those without atrophy. In a study of Japanese men, those with metastatic cancer had higher serum T levels than those with non-metastatic cancer, but high T predicted a good response to hormonal therapy [92]. Several studies have also found that in clinically localized prostate cancer a low pretreatment serum T is associated with a more advanced pathological stage and increases the probability of non-organ confined disease [93-95].

Many studies also suggest that low serum testosterone not only predicts a poor response to hormonal therapy, but also increases the risk of prostate cancer. Men with low free and total testosterone levels had a significantly higher rate of cancer than those with high serum testoserone [36]. Schatzl *et al*. found that not only did men with low testosterone have more aggressive cancers, but AR expression was also elevated in these patients [37].

On the other hand, several authors have concluded that low serum testosterone predicting more advanced disease or poorer response to therapy simply reflects the poorer health of such patients rather than indicating any causal role for testosterone in cancer development [40,41]. However, on sum the clinical evidence and our theoretical results suggest that low serum testosterone induces selection for AR overexpression, which in turn may predispose cancer to treatment-resistance.

Following the results of the PCPT, which showed an overall reduction in cancer incidence for those taking finasteride but an increase in high-grade tumors [50], great controversy has surrounded the possible role of the drug in cancer etiology [52]. It is not clear why inhibiting DHT would be protective against cancer if it is true that low serum T can promote cancer. It seems unlikely that inhibiting DHT within the prostate would have a qualitatively different effect on androgen-mediated cell activities than lowering serum T, and one recent study found that low intraprostatic DHT increased cancer aggressiveness [96].

In our model, the effect of 5-*α *reductase inhibition on selection for AR overexpression is similar to the effect of low serum T. That is, AR overexpression is selected for more strongly, but the expected time to such overexpression being observed is greater than in a normal androgen environment. Therefore, we expect 5-*α *reductase inhibition to similarly increase risk for therapy-resistant and/or aggressive prostate cancer. Indeed, as early as 1993, Martel *et al*. [79] expressed concern that the use of 5*α*-reductase inhibitors in the treatment of BPH could induce selection for therapy resistant cancer cells.

Perhaps low serum T consistently predicts both increased cancer incidence and aggressiveness because in men with lifelong low testosterone the temporal window where a normal or high androgen environment results in greater AR expression (see Figures 11 and 12) is usually passed by the time cancer is diagnosed in the latter decades of life. Finasteride treatment may shift patients to the pattern of AR evolution we have observed in low androgen environments, where the prostate-wide average *R*_*t *_remains low for a long time, but increases dramatically late in time. Therefore, in a relatively short temporal window overall cancer incidence may be reduced, but a minority of patients will cross the threshold for increased AR expression and experience a high-grade tumor. In light of the clinical data on low testosterone and cancer risk, and our own results suggesting that the temporal scale for the incidence of aggressive cancer under 5*α*-reductase inhibition is not the same as that for overall cancer incidence under normal androgen environments, long-term follow-up of patients being treated with finasteride is warranted, and the response to hormonal therapy among patients who do experience prostate cancer should be studied.

In conclusion, our theoretical study suggests that AR expression in the prostate increases uniformly with age. However, low intraprostatic androgen, whether induced by low serum T or 5*α*-reductase inhibition, can increase selection for AR overexpression, and hence increased cancer aggressiveness and treatment resistance. This overexpression may not manifest itself until relatively late in life, so while those with low intraprostatic androgen may be at an increased lifetime risk for aggressive cancer, they may experience decreased overall cancer incidence. This may help to explain the decreased overall cancer incidence, but increased risk of high-grade cancer, that has been seen in men taking finasteride [50,54].

## Competing interests

The authors declare that they have no competing interests.

## Authors' contributions

SE, JN, and YK designed the model. SE wrote the computer code and drafted the manuscript. JN and YK helped to draft the manuscript. All authors read and approved the final manuscript.

## Reviewers' Comments

### Reviewer's report 1

Dr. Ariosto S. Silva (nominated by Dr. Marek Kimmel)

1. The idea proposed in this article that there are "sweet spots" in the concentrations of hormones and in stages of development of the tumor that guide tumorigenesis and lead to therapy resistance is an important concept towards personalize cancer treatment. It reminds me of the analogy of the pepper shaker and the steak: a little pepper makes the steak taste better but a lot of pepper makes the steak a lot worse.

I would suggest rewriting this sentence for sake of clarity "Most cancers are more aggressive following HR recurrence, there are no effective treatments for such cancers, and average survival following progression does not exceed 15 months [24]." I understood that most cancers that recur and are hormone refractory are more aggressive than before treatment and that they do not respond to treatment, is this it?

Authors' response: Yes, that is exactly what was meant by this sentence. (We would assume that, by definition, any cancer that recurs in the face of androgen ablation therapy should be considered "hormone refractory.")

2. Please review these two sentences; they seem to mean the same thing: "AR amplification occurs in perhaps 30% of recurring HR tumors [22]. However, such genetic alterations do not occur in most HR cancers."

*Authors' response: Here we are pointing out that while AR gene amplification does occur and is not an insignificant contributor to HR tumor recurrence, such a genetic alteration does not occur in the majority of HR tumors. You are correct that the second sentence is somewhat redundant, but we have left it in to emphasize that this is not the dominant pathway by which HR recurrence occurs (although we did replace the wording "most HR cancers" by "the majority of HR cancers")*.

3. Please clarify how this paragraph relate to previous observation, it seems contradictory that the upregulation of AR expression is HR tumors but is also the most important pathway: "Upregulation of the AR protein is perhaps the single most important pathway by which cancers achieve androgen independence. Chen et al. [30] found that in 7 prostate cancer xenograft models, increased androgen receptor expression was the only change consistently associated with HR cancer progression."

*Authors' response: In the previous paragraph, we are pointing out that amplification of the AR gene occurs in a minority of HR tumors. Increased levels of the AR protein itself, whether achieved through gene amplification or by other pathways, seems to be the most important (or at least most common) pathway to HR tumor recurrence in the majority of tumors*.

4. Could you please give more details on this trial? The subjects had any signal of prostate tumor or the idea is that every man would submit to this treatment after a certain age? "In the Prostate Cancer Prevention Trial (PCPT), finasteride use reduced overall prostate cancer incidence, but increased the risk of high-grade cancer over 7 years [50]."

*Authors' response: The PCPT was a double-blind RCT that assigned men with no history of prostate cancer to receive finasteride (5 mg) or a placebo daily for 7 years. Biopsy was performed on the basis of serum PSA or abnormal digital rectal exam. At the end of the 7-year study period, all men who consented received a biopsy. Finasteride use reduced overall prostate cancer incidence by 24%, but increased the risk of high-grade cancer: 37% of cancers were high-grade in the treatment group versus 22% in the placebo group. These details are now included in the text*.

5. Could you please explain the relationship between a smaller prostate and the probability that a biopsy would detect cancer? We could imagine that smaller prostates would pass the rectal touch exam and thus not be biopsied? "Finastide significantly reduced the prostate size in those treated, and reduced prostate size can increase the probability of cancer detection in biopsy samples, so a detection bias could explain the increased rate of high-grade cancer."

*Authors' response: What is being discussed here is not the relationship between the prostate size and the probability of biopsy based on DRE findings (although you are certainly correct that a smaller prostate would decrease the chance of a positive rectal exam and thus a for-cause biopsy)*.

*Rather, it has been argued that given a biopsy occurs, the probability that tumor tissue is present in the biopsied tissue sample will depend on the absolute size of the prostate. If more normal epithelium is present, as in the case of placebo-treated men, the probability that tumor tissue is detected decreases. Since finasteride treatment reduces the prostate volume, a tumor of equal size is more likely to be detected in biopsy samples from a finasteride-treated man than a placebo-treated man. This has been (hopefully) clarified in the text*.

6. Please elaborate on how hypoxia may induce increase in ROS. One of the explanations for limited effect of radiation therapy in hypoxic areas of tumors in the low levels of reactive oxygen species, supposedly due to the lack of oxygen. "Hypoxia impairs aerobic respiration, and mitochondrial ROS is required for stabilization of HIF-1*α *[97]. Thus, in addition to direct effects on redox related enzymes, a low androgen environment also increases ROS levels by inducing a hypoxic environment."

Authors' response: Radiation exerts its biological effects primarily through the ionization of water:

*These and other subsequently generated radical species can react with O*_2 _*to form reactive oxygen species such as the superoxide anion and hydrogen peroxide. These species initiate further radical reactions that damage the DNA. Therefore, the presence of molecular oxygen in tumor tissue, but not necessarily the presence of pre-existing ROS, is an important determinant of the efficacy of radiation therapy*.

*In a hypoxic environment the electron transport chain is impaired and oxygen is less likely to be fully reduced, resulting in partial oxygen reduction and ROS generation. Such mitochondria generated ROS stabilizes HIF-1α and thus activates the cellular response to hypoxia. We have modified this section slightly for clarity*.

7. The sentence "that low intraprostatic AR:ligand concentrations, caused either by low serum testosterone or 5-*α *reductase inhibition, drive evolution towards elevated sensitivity to androgens in prostate epithelium, therefore increasing the risk of cancer that is resistant to hormonal therapy" might be better understood if the words "elevated sensitivity" were replaced by "smaller dependency."

Authors' response: We have replaced "elevated sensitivity to" by "decreased dependence upon."

### 0.1 Reviewer's report 2

Dr. Marek Kimmel

In the transcription factor literature, there is a significant number of papers concerning spatial organization of AR activity within the nucleus, leading to effects such as hyperspeckling (see the attached paper by van Royen *et al*. 2007 from Houtsmuller's laboratory [98]). Are there cancer-specific modifications of such organization which might have anything to do with altered expression of AR-dependent genes?

*Authors' response: In this paper, van Royen et al. present a model for AR/DNA/cofacter interaction that is modulated by binding between the N terminal and C terminal domains (N/C interactions) of the AR. Such binding appears to prevent cofactor recruitment, and occurs upon hormone ligand binding. Following ligand binding, the AR translocates to the nucleus, where it is highly mobile. Transient binding to the DNA terminates N/C interactions. This allows the C-terminal ligand binding domain (LBD) to interact with cofactors containing the FxxLF motif, leading to altered transcriptional activity*.

*It is likely that cancer-specific alterations of the AR that affect the spatial kinetics of DNA binding and cofactor recruitment play a role in prostate cancer progression and its response to treatment. For example, changes in N/C interaction might alter co-factor recruitment within the nucleus, and altered co-factor activity may be associated with HR recurrence. Farla et al*. [99]*showed that while both AR agonists and antagonists cause translocation of the AR:ligand complex to the nucleus, only agonists caused DNA binding. Mutations in the AR can cause antagonists to act as agonists, and these may act by altering N/C interaction and cofactor recruitment following binding. Farla et al. established that DNA binding kinetics for mutant ARs bound to anti-androgens were similar to the DNA binding kinetics for wild-type ARs bound to agonists. Therefore, modification of DNA binding in the spatial compartment in response to non-canonical ligands is likely one pathway by which prostate cancers become hormone refractory. van Royen et al. also found that speckling overlaps with, but is not perfectly correlated with, transcriptional activity. However, speckling does not occur without DNA binding, so the dynamics of speckling are likely a direct result of DNA binding kinetics and subsequent interactions with co-factors, etc*.

*In any case, we would argue that any alteration in the AR that affects the transcriptional response to AR:ligand binding must necessarily affect the spatial dynamics of AR activity in the nucleus. At the phenomenological level, the exact molecular mechanisms by which AR activity is changed are not as important as how the androgen environment selects for altered AR activity in general*.
